# Insights into the paracrine effects of uterine natural killer cells

**DOI:** 10.3892/mmr.2014.2626

**Published:** 2014-10-13

**Authors:** XIN GONG, YANXIA LIU, ZHENZHEN CHEN, CAI XU, QIUDAN LU, ZHE JIN

**Affiliations:** 1 Department of Reproductive Endocrinology, Dongfang Hospital of Beijing University of Chinese Medicine, Beijing 100078, P.R. China; 2 Department of Chinese Materia Medica, Beijing University of Chinese Medicine, Beijing 100102, P.R. China

**Keywords:** trophoblast invasion, spiral artery remodeling, decidua, human

## Abstract

Uterine natural killer (uNK) cells are recruited into the uterus during establishment of the implantation and placentation of the embryo, and are hypothesized to regulate uterine spiral artery remodeling and angiogenesis during the initial stages of pregnancy. Failures in uNK cell activation are linked to diseases associated with pregnancy. However, the manner in which these cells interact with the endometrium remain unknown. Therefore, this study investigated the paracrine effects of uNK cells on the gene expression profile of an endometrial epithelial and stromal cell co-culture system *in vitro*, using a microarray analysis. Results from reverse transcription-quantitative polymerase chain reaction and enzyme-linked immunosorbent assay experiments showed that soluble factors from uNK cells significantly alter endometrial gene expression. In conclusion, this study suggests that paracrine effects of uNK cells guide uNK cell proliferation, trophoblast migration, endometrial decidualization and angiogenesis, and maintain non-cytotoxicity of uNK cells.

## Introduction

In the human endometrium, uterine leukocytes undergo cyclic changes in cell number during the menstrual cycle. Uterine natural killer (uNK) cells comprise 70% of all decidual leukocytes during the secretory phase, when implantation occurs, and during early pregnancy (1,2). At the initiation of embryo implantation and placentation, uNK cells interact with the extravillous trophoblasts and fetal cells that invade the uterus, where they remove and replace the smooth muscle of maternal spiral arteries (3). This ultimately converts small, coiled vessels into wider channels that are able to provide nutrients to the developing fetus (4).

The initial contact between the blastocyst and uterus occurs through adhesion of the embryonic trophectoderm to the uterine epithelium (5). During implantation, the epithelium is said to become ‘receptive’ (6). In addition, the transformation of endometrial stromal cells from small, densely packed cells to large polygonal cells with an open vesicular nucleus is one of the characteristic features of decidualization (7). These findings suggest that endometrial epithelial and stromal cells are important in implantation and decidualization. Furthermore, a previous study demonstrated that epithelial STAT3 controlled stromal function via a paracrine mechanism (8), indicating that there is epithelial-stromal crosstalk during implantation.

Implantation marks a transition stage in pregnancy, in which the blastocyst assumes a fixed position and establishes an altered physiological interaction with the uterus. The paracrine effects of uNK cells stimulate stromal fibroblasts to produce chemokines and cytokines, which support trophoblast migration during implantation. They also upregulate interleukin (IL)-15 and IL-15Rα in stromal fibroblasts that may establish an environment for uNK cells to promote cell proliferation and recruitment into the uterus (9). There is strong evidence that implantation and early pregnancy are not a single event, and do not occur simultaneously (10). Thus, in order to investigate the effects of uNK cell paracrine signaling on these processes as a whole, a co-culture system consisting of epithelial and stromal cells was created. Furthermore, the regulation of trophoblast invasion and modification of the spiral arteries, was investigated (11).

## Materials and methods

### Ethical approval

All subjects understood and signed the informed consent form prior to participation. Experimental protocols were approved by the Ethics Committee of the Dongfang Hospital Human Ethics Committee, Beijing, China (no. 2011090201).

### Tissue collection

Decidual tissues were obtained from ten healthy females undergoing an elective termination of a normal pregnancy at between seven and eight weeks of gestation, as determined by the last menstrual period.

Endometrial tissues were collected from biopsies taken during the proliferative phase of the menstrual cycle of females undergoing laparoscopy for benign disease (Dongfang Hospital of Beijing University of Chinese Medicine, Beijing, China). The exclusion criteria were hormonal stimulation, cancerous lesions and irregular menstrual bleeding. There were six volunteers and two endometrial biopsies per volunteer were obtained. Samples from three of the females (six biopsies) were used for the for microarray experiments and samples from the remaining three females (six biopsies) were used for the reverse transcription-quantitative polymerase chain reaction and enzyme-linked immunosorbent assay experiments. Of the two samples taken from each patient, one sample was used as a control and the other was used in the experimental group.

### uNK cell isolation

uNK cells were purified as previously described (2). Briefly, decidual tissues were thoroughly washed with Ca^2+^- and Mg^2+^-free Hank’s balanced salt solution (HBSS) containing 100 U/ml penicillin and 100 g/ml streptomycin (Sigma-Aldrich, St. Louis, MO, USA), cut into fragments of 1–2 mm^3^ using two scalpels and digested for 1 h at 37°C with gentle agitation in HBSS with 0.1% (w/v) collagenase I (Gibco-BRL, Carlsbad, CA, USA). Cell suspensions were layered over Ficoll-Hypaque medium (General Electric, Fairfield, CT, USA) and centrifuged at 800 × g for 25 min. Cells at the interface were washed twice in RPMI-1640 media with 10% fetal calf serum (FCS) and antibiotics. Following incubation for 20 min at 4°C with anti-CD56 micro beads (Miltenyi Biotec GmbH, Bergisch Gladbach, Germany), cells were washed in washing buffer [phosphate-buffered saline (PBS), EDTA 2 mM and 0.5% bovine serum albumin(w/v)] and loaded onto a manual cell separation (MS) column in a MiniMACS magnet (MiniMACS^TM^ Separator System; Miltenyi Biotec GmbH). The MS column was flushed three times and CD56^+^ cells were flushed according to the manufacturer’s instructions. The purity of the uNK cells was >90% CD56^+^CD3^−^ according to flow cytometric analysis. The uNK cells were cultured in RPMI-1640 media with 1% FCS and 10 ng/ml IL-15 (R&D Systems Inc., Minneapolis, MN, USA).

### uNK cell-secretion medium production

uNK cell-secretion medium was prepared using 200 μl RPMI-1640 media with 1% FCS and IL-15 (10 ng/ml) containing 5×10^5^ of the purified uNK cells and placed into the upper chamber of a 0.4-μm pore hanging cell culture insert (EMD Millipore, Billerica, MA, USA) in a 24-well tissue plate, with 1,300 μl of the same media excluding cells in the lower chamber. Germeyer *et al* (9) showed that soluble factors from uterine leucocytes had significant effects on endometrial cell gene expression. Thus, a hanging cell culture insert was used so that soluble molecules from the uNK cells were able pass through the filter into the lower chamber, without cells being in direct contact. The control medium comprised 1,500 μl RPMI-1640 media with 1% FCS and 10 ng/ml IL-15. This was used for subsequent experiments. Following incubation for 24 h at 37°C, the uNK cell-secretion medium from the lower chamber and the control media were collected. To reduce interassay variability, the media from several batches was pooled for subsequent experiments and frozen at −80°C. Cells in the upper chamber were collected and the cell viability was measured using a live/dead viability kit (Invitrogen Life Technologies, Carlsbad, CA, USA). Only uNK cell samples containing <35% of dead cells following overnight incubation were used for subsequent experiments.

### Endometrial stromal and epithelial cell isolation

Human endometrial tissue was dissociated into single cells using 0.1% (w/v) collagenase I (Life Technologies, Carlsbad, CA, USA) for 50–60 min at 37°C. Cell suspensions were filtered using a 40-μm sieve to separate undigested myometrial tissue and debris. Further dissociation of the filtrate was prevented by Dulbecco’s modified Eagle’s medium (DMEM)/F-12 (no Phenol Red; Gibco-BRL) with 10% FBS (Gibco-BRL). To remove erythrocytes, the cells were resuspended in 4 ml DMEM/F12 with 1% FCS, layered over Ficoll-Paque PLUS (General Electric) and centrifuged for 25 min at 800 × g. Endometrial cells were removed from the Ficoll-Paque PLUS medium interface, washed three times and resuspended in 1 ml DMEM/F12 with 1% FCS. Leukocytes were removed with CD45-coated Dynabeads (Invitrogen Life Technologies). Purified stromal and epithelial cell suspensions were then obtained by a further round of magnetic bead sorting using Collection Epithelial Enrich Dynabeads (Invitrogen Life Technologies). Epithelial and stromal cell preparations were >95% pure.

Stromal cells were cultured in DMEM/F12 with 10% FBS and an antibiotic-antimycotic agent (100 U/ml penicillin, 100 g/ml streptomycin, 10 μg/ml gentamicin 0.25 μg/ml amphotericin B; Life Technologies). Epithelial cells were cultured in serum-free bronchial epithelial cell growth medium (final volumes: 2 ml bovine pituitary extract, 0.5 ml insulin, 0.5 ml HC, 0.5 ml GA-1000, 0.5 ml retinoic acid, 0.5 ml transferrin, 0.5 ml triiodothyronine, 0.5 ml epinephrine and 0.5 ml hEGF; Lonza, Walkersville, MD, USA) and an antibiotic-antimycotic agent. The isolated stromal and epithelial cells were separately seeded into six-well plates with 3 ml culture medium per well. Each well contained stromal or epithelial cells from a single patient. After two weeks, cells were passaged into 25 cm^2^ cell culture flasks. Following this, stromal cells were passaged every 4–5 days and epithelial cells were passaged every 9–10 days.

### Co-culture system

On day 20 following endometrial stromal and epithelial cell generation, cells of each type were seeded onto a Nunc UpCell Surface membrane (Thermo Labsystems, Santa Rosa, CA, USA). The co-culture system was built on these temperature-responsive cell culture surfaces, according to the manufacturer’s instructions. In brief, when cells reached 80% confluence, all medium was aspirated and 500 μl fresh medium was added. The membrane was then placed on top of the stromal cell layer. The Nunc UpCell Surface was maintained at 20°C for 13 min. The membrane and cell layer were then carefully removed from the Nunc UpCell Surface using forceps. The membrane with the attached cell layer was transferred facing downwards onto the epithelial cell surface. Fresh medium was added and samples were incubated at 37°C for 40 min. A further 1 ml of medium was added to the top of the membrane and the membrane was withdrawn from the cell layer. The ratio of stromal to epithelial cells was 1:1, and every co-culture system was built using stromal and epithelial cells from the same participant. Co-cultured cells were maintained in DMEM/F12 with 1% FCS.

### Co-culture system treatment

Each group, control and uNK cell, contained six co-culture systems. All the groups were washed twice with PBS and placed in serum-free DMEM for 16 h prior to subsequent experiments. DMEM was replaced by 80% uNK cell-secretion medium and 20% DMEM in the uNK cell group, whilst the control group was treated with 80% control medium and 20% DMEM. Following incubation for 6 h, the cells and media from each group were collected. Three pairs of co-culture systems were used for the microarray studies and three pairs for the RT-qPCR experiments.

### Microarray experiments

Total RNA was extracted from the endometrial cells in each co-culture system. RNA was purified with the RNeasy Mini kit (Qiagen, Hilden, Germany), according to the manufacturer’s instructions. The microarray analysis was performed using the GeneChip^®^ 3′ IVT Express kit (Affymetrix Inc., Santa Clara, CA, USA). Briefly, total RNA underwent reverse transcription, first strand cDNA synthesis, double strand DNA, *in vitro* transcription, cRNA synthesis and fragmentation (12). Samples were hybridized onto GeneChip PrimeView Human Gene Expression Array (Affymetrix Inc.). This array covers >36,000 transcripts and variants. Following 16 h hybridization at 45°C, arrays were washed on Fluidics Station 450 (Affymetrix, Inc.) and were scanned with Scanner 3000 (Affymetrix, Inc.) in order to obtain quantitative gene expression levels. The control and uNK cell groups were processed simultaneously throughout. Three chips were analyzed for each group.

### RT-qPCR analysis

A total of four differentially expressed genes were selected for validation of the results from the microarray experiments using RT-qPCR. Cells from the control and uNK cell groups were washed twice with PBS, and total RNA was extracted using TRIzol (Life Technologies). Reverse transcription was performed with 8 μl of total RNA per 20 μl reaction using a standard cDNA Synthesis kit (Takara Bio, Inc., Otsu, Japan). The RT-qPCR primer sequences for target genes were self-designed by this group and ordered from Invitrogen. Primer sequences for target genes are shown in Table I.

For each RT-qPCR experiment, the typical thermal cycling conditions included an initial activation step at 95°C for 5 min, 40 cycles at 95°C each for 30 sec, 56°C for 20 sec and 72°C for 30 sec. PCR reactions were performed on ABI Prism 7700 Sequence Detection system (Applied Biosystems Life Technologies, Foster City, CA, USA). cDNA concentration was normalized to that of GAPDH. The target mRNA expression was analyzed using the 2^−ΔΔCt^ algorithm.

### ELISA experiments

IL-15 was analyzed using a commercially available ELISA kit (ELH-IL-15, RayBiotech, China) in the uNK cell-secretion medium prior to its use in the co-culture system experiments and in supernatants of the co-culture systems that had been treated with control media or with uNK cell-secretion medium. The analysis was conducted according to the manufacturer’s instructions. Assays were performed in triplicate and concentrations of IL-15 (pg/ml) were compared with standard curves. To determine the quantities of IL-15 secreted by the co-culture systems, the starting IL-15 content of the uNK cell-secretion medium was subtracted. The sensitivity of the kit was 10 pg/ml.

### Statistical analysis

Data were analyzed using analysis of variance using SPSS 17.0 software (SPSS, Inc., Chicago, IL, USA). In the microarray experiments, median fold change ratios between the control and uNK cell groups were derived for each transcript, and genes that were up- or downregulated with a fold change >1 and P<0.001 were selected. In RT-qPCR and ELISA analysis, data are presented as the median ± standard error of the mean. P<0.01 was considered to indicate a statistically significant difference. Graphs of the data were produced using Microsoft Excel software.

## Results

### Microarray experiments

Gene expression profiling using a microarray was used to compare transcript expression in the endometrial co-culture systems treated with either control medium or uNK cell-secretion medium.

This analysis identified 155 upregulated genes that exhibited a change of >2-fold in the median expression level in response to uNK cell-secretion medium. No transcript was downregulated >2-fold (Table II). However, certain genes in uNK cell groups were upregulated with a 1.0–2.0-fold change, compared with the control group. Previous studies have shown that these genes have an important functions in uNK cells. For instance, the co-culture system treated with uNK cell-secretion medium showed increased expression of interleukin (IL)15RA (1.6-fold) (13), vascular endothelial growth factor (VEGF)-C (1.35-fold) (14), intercellular adhesion molucule (ICAM) 1 (1.66-fold) (15), superoxide dismutase (SOD)2 (1.09-fold), caspase (CASP)1 (1.85-fold), nuclear factor erythroid 2-related factor 3 (NFE2L3) (1.37-fold), interferon γ receptor 1 (IFNGR1; 1.10-fold) (16), major histocompatibility complex (MHC) class I polypeptide-related sequence A (1.12-fold) and MHC class I polypeptide-related sequence B (1.34-fold) (3,17) and showed decreased expression of IGFBP3 (1.04-fold) (P<0.001).

### RT-qPCR analysis

**Figure 1:**
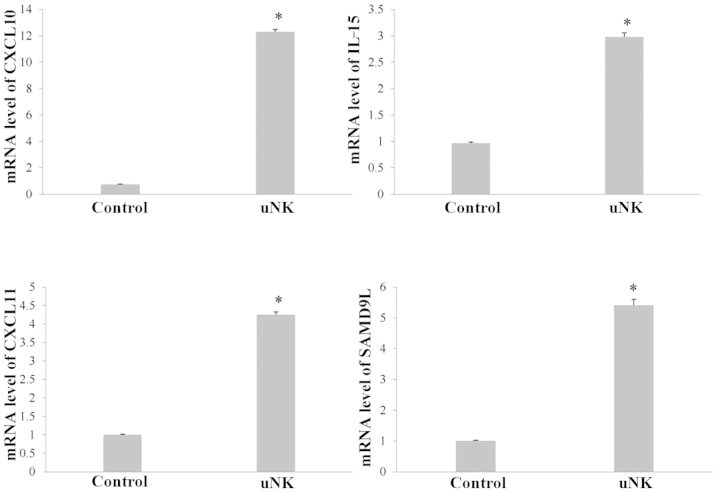
Transcript levels of CXCL10, CXCL11, IL-15 and SAMD9L determined by RT-qPCR. The RT-qPCR results were consistent with those of the microarray analysis. Data are expressed as the mean ± standard error of the mean. ^*^P<0.01, compared with the control group. uNK, uterine natural killer cells; CXCL, chemokine (C-X-C) motif ligand; IL-15, interleukin 15; SAMD9L, sterile α motif domain containing 9-like; RT-qPCR, reverse transcription-quantitative polymerase chain reaction.

In order to verify these changes, transcript levels for certain genes were measured by RT-qPCR, including chemokine (C-X-C) motif ligand (CXCL)10, CXCL11, IL-15 and sterile α motif domain containing 9-like (SAMD9L; Fig. 1). The RT-qPCR results confirmed the significant changes in expression that had been indicated by the microarray analysis. The endometrial co-culture system treated with uNK cell-secretion medium showed increased expression of CXCL10 (16.4-fold), CXCL11 (4.3-fold), IL-15 (3.1-fold) and SAMD9L (5.4-fold; P<0.01).

### ELISA experiments

**Figure 2:**
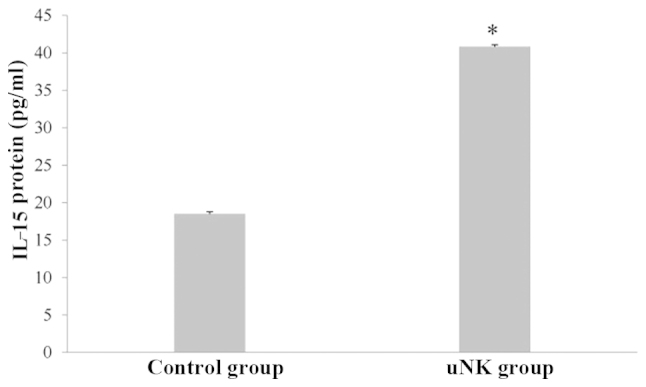
IL-15 levels in control and uNK groups. An enzyme-linked immunosorbent assay was used to measure IL-15 levels in supernatants from co-culture systems treated for 6 h with conditioned medium from decidual uNK cells or with control medium. Data are presented as the mean ±standard error of the mean. Values were corrected for the quantity of IL-15 detected in the control and uNK cell-secretion medium prior to addition to the co-culture systems. ^*^P<0.01, compared with the control group. IL-15, interleukin 15; uNK, uterine natural killer cells.

To confirm the observed changes in the IL-15 protein level, the quantity of IL-15 was analyzed by ELISA in the supernatant of the control and uNK cell-secretion medium-stimulated endometrial co-culture systems. There was a significant increase in IL-15 protein levels in the experimental groups compared with the control group (Fig. 2; P<0.01).

## Discussion

Several observations suggest that uNK cells are involved in reproduction. They increase in number during the luteal period of the menstrual cycle when implantation occurs (1). They are present in the early phases of gestation, when placental cells invade into the maternal arteries (18). In addition, they are particularly abundant in the area surrounding the infiltrating fetally derived extravillous cells (19). During the progesterone-dominated phase of the menstrual cycle, uNK cells show changes in the levels of transcripts for VEGF-C (20). Previous protein array studies have shown that uNK cells are the predominant producers of angiogenic growth factors in early pregnancy (21). In addition, in an *ex vivo* chorionic plate artery model, uNK cells promoted vessel-like assembly of extravillous cytotrophoblast cell lines (15,22). Insufficient uNK cell activation may reduce these processes and contribute to poor arterial remodeling in decidua, thus increasing the risk of preeclampsia and intrauterine fetal growth restriction (23).

In humans, CD56^+^ NK cells are associated with the synthesis of immunoregulatory cytokines, particularly IFN-γ (24). IFN-γ significantly upregulates certain chemokines [CXCL9, CXCL10, chemokine (C-C motif) ligand 8 and IL-15Rα], enzymes [guanylate binding protein 5, transporter associated with antigen processing (TAP1), SOD2 and CASP1] and transcription factors (interferon regulatory factor 1, NFE2L3 and transcription factor AP-2 γ). It is also known to downregulate insulin-like growth factor binding proteins (Wnt1 inducible signaling pathway 2 and insulin-like growth factor-binding protein 3) (16). These actions, combined with the uNK cell production of chemokines CXCL10 and CXCR2, direct the migration and invasion of trophoblasts (25) and promote angiogenesis in the placental bed (26,27). The present study found similar changes in gene expression, for example, IFNGR1 transcript levels were significantly increased in co-culture systems stimulated by uNK cell-secretion medium. This concordance strongly suggests that uNK cell paracrine signaling, combined with INF-γ, regulates the expression of genes involved in embryo and trophoblast migration, endometrial decidualization and angiogenesis in human uterine endometrium.

Implantation-associated decidualization in the rat and mouse results in the accumulation of NK cells in the uterine mesometrial decidua (28). uNK cells are hypothesized to be involved in pregnancy-associated uterine vascular development (20). However, it is not clear how uNK cells communicate with the developing endometrial cells in order to facilitate this process. Previous *in vitro* experimentation has indicated that uNK cells produce factors that directly affect the behavior of trophoblast cells (25). A number of studies have suggested that uNK cell supernatant stimulate trophoblast invasion (29), whereas others have concluded that it is the uNK cell supernatants that stimulate these process (20). The results of the current study indicate that the paracrine effects of uNK cells on endometrial epithelial and stromal cells mediate the development of the vasculature. Levels of ICAM-1, which is involved the migration and network formation of the trophoblast cell line (15), increased significantly in this study. Thus, it is likely that ICAM-1 is involved in this paracrine network.

Although replete with cytotoxic machinery, uNK cells remain tolerant at the maternal-fetal interface (26). A previous indicated that this is facilitated by VEGF-C (30), and *in vitro* studies have also suggested that the involvement of IL-15 is important in promoting this tolerance (17). In addition, the non-cytotoxic capacity of uNK cells is based on their ability to recognize surface MHC class I molecules on target cells, which deliver signals that suppress NK cell function. A number of studies have suggested that a lack of engagement of MHC-specific receptors leads to NK cell-mediated killing (17,31). The results from this study showed that there was an increased expression of VEGF-C, IL-15 and MHC class I polypeptide-related sequence A/B in the co-culture systems that were treated with uNK cell-secretion medium compared with the control group, indicating similar non-cytotoxic mechanism to those already postulated. Furthermore, there is evidence that endothelial cells exhibit sensitivity to activated peripheral blood NK cells in the absence of the expression of TAP1 (32), as TAP-1 is a key factor essential for peptide loading for MHC class I assembly (33). It is proposed that VEGF-C is the predominant regulator of TAP1 expression in the uterus (30), and the present study showed higher TAP1 expression in the uNK cell group. These findings support a dual function of VEGF-C, in which it acts as an angiogenic factor and also promotes immune tolerance in the uterine microenvironment. To the best of our knowledge, this is the first evidence that noncytotoxicity of uNK cells is directly coupled to their vascular remodeling and angiogenesis functions.

The origins of human uNK cells are not clear, and a number of mechanisms have been postulated (34–36). Several of the molecules that were altered by uNK cell-secretion medium in this study are known to have important functions in NK cell proliferation, including IL-15 and IL-15Rα. IL-15 has a variety of functions, including the induction of T cell proliferation and the activation of cytotoxic effector cells and monocytes (37,38). The reciprocal interactions between uNK cells and the epithelial/stromal cell co-culture system observed in this study are similar to those in bone marrow during NK cell development. NK cells upregulate IL-15 in the bone marrow microenvironment, which is then bound and presented by IL-15Rα to the stromal cell surface, promoting increased NK cell proliferation (39). These results suggest that this mechanism may occur in the endometrium in response to molecules secreted by uNK cells. The results suggest that uNK cells and non-decidualized stromal and epithelial cells may interact to maintain immune cell homeostasis in the endometrial microenvironment.

In conclusion, to the best of our knowledge, this is the first detailed study of the paracrine interaction between uNK cells and endometrial cells (epithelial/stromal cells). It indicates that this paracrine signaling may contribute to uNK cell proliferation and recruitment, embryo and trophoblast migration, endometrial decidualization, angiogenesis and immune tolerance in the uterine microenvironment.

## Figures and Tables

**Table I:** Sequences of primers for reverse transcription-quantitative polymerase chain reaction.

Gene	Primer sequence 5′g3′	Length (bases)	Amplicon (bp)
CXCL10	F: CTTTCTGACTCTAAGTGGCATTC	23	176
	R: CACCCTTCTTTTTCATTGTAGCAA	24	
CXCL11	F: TATTACTATCTGTGGTTACGGTGGAG	26	269
	R: GCACTTTTGCCAGTATCCCAT	21	
IL-15	F: TGGCTGCTGGAAACCC	16	123
	R:CACAAGTAGCACTGGATGGAAAT	23	
SAMD9L	F: GCCTTATCTCCACCTGTTTCTTAG	24	300
	R: TGGGATGGCATTCCTTGAC	19	
GAPDH	F: GAGCCAAAAGGGTCATCATCT	21	231
	R: AGGGGCCATCCACAGTCTTC	20	

F, forward; R, reverse; CXCL, chemokine (C-X-C) motif ligand; IL-15, interleukin-15; SAMD9L, sterile α motif domain containing 9-like.

**Table II:** Upregulated transcripts altered >2-fold.

Gene	Fold change	Gene ID	Description
Cytokines/ Chemokines			
CXCL10	11.1	3627	Chemokine (C-X-C motif) ligand 10
CXCL11	4.2	6373	Chemokine (C-X-C motif) ligand 11
IL-15	2.6	3600	Interleukin 15
IL-7	2.1	3574	Interleukin 7
Immunological factors			
NLRC5	3.8	84166	NLR family, CARD domain containing 5
FAM111A	3.4	63901	Family with sequence similarity 111, member A
ACTR2	2.6	10097	ARP2 actin-related protein 2 homolog (yeast)
HSPH1	2.2	10808	Heat shock protein 1
IFIT5	2.2	24138	Interferon-induced protein with tetratricopeptide repeats 5
UVRAG	2.1	7405	UV radiation resistance associated gene
Apoptotic protein			
RASSF6	2.2	166824	Ras association (RalGDS/AF-6) domain family member 6
Tryptophan metabolism			
IDO1	3.6	3620	Indoleamine 2,3-dioxygenase 1
Signaling factors			
GBP2	3.2	2634	Guanylate binding protein 2, interferon-inducible
UACA	3.1	55075	Uveal autoantigen with coiled-coil domains and ankyrin repeats
IFIT3	2.8	3437	Interferon-induced protein with tetratricopeptide repeats 3
ALCAM	2.8	214	Activated leukocyte cell adhesion molecule
CGA	2.6	1081	Glycoprotein hormones, α polypeptide
EDNRA	2.5	1909	Endothelin receptor type A
WASF2	2.2	10163	WAS protein family, member 2
IL6ST	2.1	3572	Interleukin 6 signal transducer
USP15	2.1	9958	Ubiquitin specific peptidase 15
MIER1	2.1	57708	Mesoderm induction early response 1 homolog
RGS12	2.0	6002	Regulator of G-protein signaling 12
Transcription			
STAT1	3.7	6772	Signal transducer and activator of transcription 1
IRF1	3.6	3659	Interferon regulatory factor 1
IRF9	3.2	10379	Interferon regulatory factor 9
IFI16	3.2	3428	Interferon, γ-inducible protein 16
ATRX	3.1	546	Alpha thalassemia/mental retardation syndrome X-linked
TCERG1	2.5	10915	Transcription elongation regulator 1
ZNF644	2.4	84146	Zinc finger protein 644
ZEB1	2.2	6935	Zinc finger E-box binding homeobox 1
SAFB2	2.1	9667	Scaffold attachment factor B2
PRDM2	2.1	7799	PR domain containing 2
Nucleotide metabolism			
NUFIP2	3.0	57532	Nuclear fragile X mental retardation protein interacting protein 2
PAPOLA	2.2	57532	Poly(A) polymerase alpha
EIF4G1	2.3	10914	Eukaryotic translation initiation factor 4γ, 1
CRCP	2.5	1981	CGRP receptor component
AGGF1	2.6	27297	Angiogenic factor with G patch and FHA domains 1
XRN2	2.1	55109	5′-3′ exoribonuclease 2
Enzyme activity			
GBP4	6.8	115361	Guanylate binding protein 4
GBP5	4.8	115362	Guanylate binding protein 5
INPP4B	4.8	8821	Inositol polyphosphate-4-phosphatase, type II
GBP7	4.7	388646	Guanylate binding protein 7
SETD2	4.2	29072	SET domain containing 2
GBP1	3.9	2633	Guanylate binding protein 1
PSMB9	3.3	5698	Proteasome (prosome, macropain) subunit, β type, 9
WASL	3.3	8976	Wiskott-Aldrich syndrome-like
PUS7L	3.0	83448	Pseudouridylate synthase 7 homolog-like
C1R	2.9	715	Complement component 1
TAP1	2.8	6890	Transporter 1, ATP-binding cassette, sub-family B (MDR/TAP)
FAF2	2.7	23197	Fas associated factor family member 2
UBE2L6	2.7	9246	Ubiquitin-conjugating enzyme E2L 6
PARP14	2.7	54625	Poly (ADP-ribose) polymerase family, member 14
PARP9	2.7	83666	Poly (ADP-ribose) polymerase family, member 9
PSME4	2.5	23198	Proteasome (prosome, macropain) activator subunit 4
OTUD4	2.5	54726	OTU domain containing 4
BIRC6	2.4	57448	Baculoviral IAP repeat containing 6
ARHGAP21	2.4	57584	Rho GTPase activating protein 21
DTX3L	2.4	151636	Deltex 3-like
RARRES3	2.3	5920	Retinoic acid receptor responder 3
MGEA5	2.3	10724	Meningioma expressed antigen 5
DCAF8	2.3	50717	DDB1 and CUL4 associated factor 8
GPD2	2.3	2820	Glycerol-3-phosphate dehydrogenase 2
UHMK1	2.3	127933	U2AF homology motif (UHM) kinase 1
PDP1	2.2	54704	Pyruvate dehyrogenase phosphatase catalytic subunit 1
USP10	2.2	9100	Ubiquitin specific peptidase 10
COIL	2.2	8161	Coilin
UFL1	2.2	23376	UFM1-specific ligase 1
USP1	2.1	7398	Ubiquitin specific peptidase 1
G2E3	2.1	55632	G2/M-phase specific E3 ubiquitin protein ligase
NF1	2.0	4763	Neurofibromin 1
PHACTR2	2.0	9749	Phosphatase and actin regulator 2
Transporters			
TPR	4.4	7175	Translocated promoter region, nuclear basket protein
APOL6	3.2	80830	Apolipoprotein L, 6
GCC2	2.8	9648	GRIP and coiled-coil domain containing 2
APOL3	2.4	80833	Apolipoprotein L, 3
CPNE3	2.4	8895	Copine III
USO1	2.2	8615	USO1 vesicle docking protein homolog
SLC38A1	2.2	81539	Solute carrier family 38, member 1
NIPAL1	2.1	152519	NIPA-like domain containing 1
KIAA1033	2.1	23325	KIAA1033
NUPL1	2.1	9818	Nucleoporin like 1
Structural factors			
MIS18BP1	3.9	55320	MIS18 binding protein 1
CDC27	3.3	996	Cell division cycle 27 homolog
CALD1	3.1	800	Caldesmon 1
LIMA1	3.0	51474	LIM domain and actin binding 1
SLMAP	2.7	7871	Sarcolemma associated protein
TLN1	2.7	7094	Talin 1
EZR	2.5	7430	Ezrin
ITGB1	2.5	3688	Integrin, β1
APPL1	2.5	26060	Adaptor protein, phosphotyrosine interaction, PH domain and leucine zipper containing 1
RTP4	2.5	64108	Receptor (chemosensory) transporter protein 4
WAPAL	2.4	23063	Wings apart-like homolog
PRRC2C	2.4	23215	Proline-rich coiled-coil 2C
CASC5	2.4	57082	Cancer susceptibility candidate 5
ENC1	2.3	8507	Ectodermal-neural cortex 1
KIF14	2.2	9928	Kinesin family member 14
SPTBN1	2.2	6711	Spectrin, β, non-erythrocytic 1
MYO6	2.2	4646	Myosin VI
ODF2L	2.1	57489	Outer dense fiber of sperm tails 2-like
DYNC1H1	2.1	1778	Dynein, cytoplasmic 1, heavy chain
PPP4R2	2.1	151987	Protein phosphatase 4, regulatory subunit 2
SRPR	2.0	6734	Signal recognition particle receptor
Kinase			
WNK1	4.0	65125	WNK lysine deficient protein kinase 1
PRKDC	3.5	5591	Protein kinase, DNA-activated, catalytic polypeptide
IQGAP1	3.0	8826	IQ motif containing GTPase activating protein 1
CMPK2	2.8	129607	Cytidine monophosphate (UMP-CMP) kinase 2, mitochondrial
BAZ1B	2.7	9031	Bromodomain adjacent to zinc finger domain, 1B
CCND2	2.6	894	Cyclin D2
MOB1A	2.5	55233	MOB kinase activator 1A
MAP4K5	2.5	11183	Mitogen-activated protein kinase kinase kinase kinase 5
Ion binding proteins			
DSC2	4.5	1824	Desmocollin 2
EEA1	4.2	8411	Early endosome antigen 1
ZC3H11A	3.3	9877	Zinc finger CCCH-type containing 11A
CLSTN1	2.8	22883	Calsyntenin 1
C1S	2.7	716	Complement component 1, s subcomponent
THAP6	2.6	152815	THAP domain containing 6
RSAD2	2.5	91543	Radical S-adenosyl methionine domain containing 2
ZNFX1	2.3	57169	Zinc finger, NFX1-type containing 1
PLCB4	2.2	5332	Phospholipase C, β4
TIPARP	2.2	25976	TCDD-inducible poly(ADP-ribose) polymerase
WDFY1	2.1	57590	WD repeat and FYVE domain containing 1
CHURC1	2.1	91612	Churchill domain containing 1
ITGA4	2.1	3676	Integrin, α4
XAF1	2.1	54739	XIAP associated factor 1
DNA/RNA proteins			
ZFR	4.4	51663	Zinc finger RNA binding protein
TOP1	4.2	7150	Topoisomerase (DNA) I
BOD1L1	3.4	259282	Biorientation of chromosomes in cell division 1-like 1
IFIT2	3.3	3433	Interferon-induced protein with tetratricopeptide repeats 2
CENPF	3.1	1063	Centromere protein F, 350/400 kDa (mitosin)
MBNL1	3.0	4154	Muscleblind-like splicing regulator 1
DHX9	2.4	1660	DEAH (Asp-Glu-Ala-His) box polypeptide 9
FMR1	2.4	2332	Fragile X mental retardation 1
DDX58	2.3	23586	DEAD (Asp-Glu-Ala-Asp) box polypeptide 58
BCLAF1	2.3	9774	BCL2-associated transcription factor 1
DNMT1	2.2	1786	DNA (cytosine-5-)-methyltransferase 1
NOL8	2.2	55035	Nucleolar protein 8
RNF213	2.2	57674	Ring finger protein 213
BDP1	2.2	55814	B double prime 1
RAD21	2.2	5885	RAD21 homolog
HP1BP3	2.1	50809	Heterochromatin protein 1, binding protein 3
SF3B1	2.0	23451	Splicing factor 3b, subunit 1
Other			
SAMD9L	5.7	219285	Sterile α motif domain containing 9-like
C10orf118	5.5	55088	Chromosome 10 open reading frame 118
MTUS1	2.7	57509	Microtubule associated tumor suppressor 1
EPSTI1	2.6	94240	Epithelial stromal interaction 1
ATXN7L3B	2.6	552889	Ataxin 7-like 3B
ANKRD32	2.5	84250	Ankyrin repeat domain 32
EHBP1	2.4	23301	EH domain binding protein 1
PPFIBP1	2.4	8496	PTPRF interacting protein, binding protein 1 (liprin β1)
TMTC3	2.3	160418	Transmembrane and tetratricopeptide repeat containing 3
CEP350	2.2	9857	Centrosomal protein 350 kDa
BTBD10	2.2	84280	BTB (POZ) domain containing 10
BTN3A3	2.1	10384	Butyrophilin, subfamily 3, member A3
KCTD9	2.1	54793	Potassium channel tetramerisation domain containing 9

Gene transcription in co-culture systems following stimulation with uNK cell-secretion medium using a GeneChip PrimeView Human Gene Expression Array. P<0.001, compared with control group. Upregulated genes: 155 transcripts. uNK, uterine natural killer cells.
